# Public health applications of historical smoke forecasts: An evaluation of archived BlueSky data for the coterminous United States, 2015–2018

**DOI:** 10.1016/j.cageo.2022.105267

**Published:** 2023

**Authors:** Ryan Michael, Maria C. Mirabelli, Ambarish Vaidyanathan

**Affiliations:** a Oak Ridge Institute for Science and Education, P.O. Box 117, Oak Ridge, TN, 37831-0117, USA; b Climate and Health Program, National Center for Environmental Health, Centers for Disease Control and Prevention, 4770 Buford Highway NE, Mailstop S106-6, Atlanta, GA, 30341, USA; c Asthma and Community Health Branch, National Center for Environmental Health, Centers for Disease Control and Prevention, 4770 Buford Highway NE, Mailstop S106-6, Atlanta, GA, 30341, USA

**Keywords:** Air quality, BlueSky, Forecast, PM_2.5_, Population exposure, Wildfire smoke

## Abstract

**Background:**

Wildfires are increasing in magnitude, frequency, and severity. Populations in the wildland-urban interface and in downwind communities are at increased risk of exposure to elevated concentrations of fine particulate matter (PM_2.5_) and other harmful components of wildfire smoke. We conducted this analysis to evaluate the use of modeled predictions of wildfire smoke to create county-level measures of smoke exposure for public health research and surveillance.

**Methods:**

We evaluated four years (2015–2018) of grid-based North American Mesoscale (NAM)-derived PM_2.5_ forecasts from the U.S. Forest Service BlueSky modeling framework with monitoring data from the Environmental Protection Agency Air Quality System (AQS), the Interagency Monitoring of Protected Visual Environments (IMPROVE), the Western Regional Climate Center (WRCC), and the Interagency Real Time Smoke Monitoring (AIRSIS) programs. To assess relationships between model-derived estimates and monitor-based observations, we assessed Spearman’s correlations by spatial (i.e., county, level of urbanization, states in the western United States impacted by major wildfires, and climate regions) and temporal (i.e., month and wildfire activity periods) characteristics. We then generated county-level smoke estimates and examined spatial and temporal patterns in total and person-days of smoke exposure.

**Results:**

Across all counties in the coterminous United States and for all days, the correlation between county-level model- and monitor-derived PM_2.5_ estimates was 0.14 (p < 0.001). Correlations were stronger using data from temporary monitors and for areas and days impacted by high wildfire smoke, especially in the western United States. Correlations between county-level model- and monitor-derived estimates in non-metropolitan counties, and at higher concentrations ranged from 0.25 to 0.54 (p < 0.001).

**Conclusions:**

In general, public health practitioners and health researchers need to consider the pros and cons associated with modeled data products for conducting health analyses. Our results support the use of model-derived smoke estimates to identify communities impacted by heavy smoke events, especially during emergency response and for communities located near wildfire episodes.

## 1. Introduction

Wildfires produce smoke plumes that impact local, regional, and global air quality. The extant scientific literature confirms an association between exposure to smoke, specifically fire-related PM, and all-cause mortality and respiratory morbidity (Alman et al., 2016; Liu et al., 2015, 2017; Zu et al., 2016). Effective characterization of exposed populations and understanding health risks associated with exposure to wildfire-related PM require the availability of reliable environmental data that describe the geographic location of wildfire events, pollutant concentrations observed, and the duration of exposure. Currently, the characterization of ground-level smoke concentrations across the United States is achieved by a combination of permanent air quality monitoring networks and temporarily deployed air quality monitors. However, these monitoring networks are not designed to track population-level exposures at continuous spatiotemporal scales, with studies indicating an inability of permanent monitors to adequately characterize population smoke exposure, especially in rural areas (Jaffe et al., 2020).

To fill this data gap, satellite retrievals and atmospheric models are used to estimate ambient PM ≤ 2.5 μm in aerodynamic diameter (PM_2.5_) levels in areas with no direct measurements. Additionally, researchers have developed and evaluated smoke forecasting systems that couple fire detection systems, fuel characteristics from land cover data, and emissions modeling to estimate current and short-term smoke forecasts (Draxier and Hess, 1998; Herron-Thorpe et al., 2012; Larkin et al., 2009; Stein et al., 2015; Vaughan et al., 2004; Castrillón et al., 2011; Ferreira et al., 2020). However, little work has been done to assess the use of these forecast systems for public health surveillance and research.

This paper describes the evaluation of North American Mesoscale (NAM)-based modeled predictions of surface PM_2.5_ concentrations from the U.S. Forest Service (USFS) BlueSky modeling framework using existing observation data from the Environmental Protection Agency Air Quality System (AQS), the Interagency Monitoring of Protected Visual Environments (IMPROVE), the Western Regional Climate Center (WRCC), and the Interagency Realtime Smoke Monitoring program (AIRSIS) monitoring programs. In the following sections, we briefly describe the BlueSky smoke data product, discuss methods used to retrieve and process the smoke data, describe methods for the generation of population-level smoke exposure metrics, and evaluate model-derived estimates using PM_2.5_ measurements from the AQS, IMPROVE, WRCC, and AIRSIS observation networks. Finally, we discuss the applicability of archived smoke forecasts for creating population-level exposure metrics at the county level.

## 2. Materials and methods

### 2.1. BlueSky modeling framework

BlueSky is a modular framework for modeling the emissions, transport, and chemistry of smoke from wildland fires (Larkin et al., 2009). The BlueSky framework links together several models describing fire information (e.g., fire location, fire size, fire type), fuel loading (e.g., fuel type and amount), fuel consumption, speciated emissions, emissions dispersion, and emissions trajectories. Three-dimensional gridded meteorological data required for trajectory and dispersion calculations are provided by a variety of models including the National Center for Atmospheric Research Mesoscale Meteorological model, and the more recent Weather Research and Forecasting model. Simulations of trajectories are performed using the Hybrid Single-Particle Lagrangian Integrated Trajectory model. Details about the overall modeling framework, the component models, and how they are linked together are described in Larkin et al. (2009). Daily BlueSky predictions of surface PM_2.5_ concentrations from wildfires are available across the coterminous United States through the USFS in support of the Interagency Wildland Fire Air Quality Response Program.

For our evaluation, we downloaded BlueSky daily archived model forecasts from AirFire’s FTP server (U.S. Forest Service, 2020) for the years 2015 through 2018. The data have a spatial resolution of 4 km and a temporal resolution of 1 h. The daily forecast records contain either 24 h, 48 h, or 72 h of forecast smoke data. We selected the most recent forecast data hour from the daily forecast records for all analyses performed here. Additionally, we used forecast dispersion records that incorporated carry-over smoke, that is, predictions of smoke leftover from the previous forecast period carried into the current forecast period. The hourly resolved arrays were adjusted for local standard time, then aggregated to create daily county-level summaries of mean surface PM_2.5_ concentration for the coterminous United States over the period January 1, 2015 through December 31, 2018.

### 2.2. Surface observation data

To evaluate the model-derived data, we used measurements from permanent monitors in the AQS and IMPROVE monitoring networks and temporary monitors in the WRCC and AIRSIS monitoring programs. For the permanent network data, we downloaded 24-h average PM_2.5_ observations, using records from January 1, 2015 through December 31, 2018. For the IMPROVE data, we used the observed elemental carbon fraction of total PM_2.5_ mass to evaluate the forecast data. The AQS and IMPROVE monitoring networks are maintained by state environmental agencies and the data are publicly available (U.S. Environmental Protection Agency, 2019; IWFQRP, 2020) For the temporary network data, we obtained hourly averaged PM_2.5_ observations, which were adjusted for local standard time and aggregated to create county-level daily summaries of mean PM_2.5_ concentrations. The WRCC is one of six regional climate centers in the United States, is administered by National Oceanic and Atmospheric Administration (WRCC, 2000), and the AIRSIS program is administered through the U.S. Forest Service (U.S. Forest Service, 2013).

### 2.3. Generation of county-level smoke estimates

We used a population-weighted county centroid containment approach to map grid cells to counties and assign daily summaries of grid-level PM_2.5_ forecast to the county level (Vaidyanathan et al., 2013). Hourly smoke forecasts from the model are available at a 4 km-by-4km grid resolution. We used the hourly forecast data in a multi-stage geo--imputation procedure to convert grid-level smoke forecast data to county-level estimates. We first assigned each U.S. Census block centroid to a forecast-derived grid cell based on a containment relationship and created block-level estimates of hourly smoke forecasts. Using block-level population as weights, we then calculated a population-weighted average of daily 24-h mean smoke predictions by U.S. Census tracts. From this Census tract-level data product, we created average county-level estimates of daily 24-h mean smoke forecasts using tract population as weights.

### 2.4. Analysis: evaluation of county-level estimates

To compare the temporal variability in county-level estimates for the datasets, we created time series of the daily summaries of population-weighted county-level concentrations for our domain, aggregating the summaries by year, month, and wildfire activity periods. We defined wildfire activity periods as March 1–June 30 and July 1–October 31. We evaluated the model-derived estimates against PM_2.5_ observations from the monitoring networks over the period 2015 through 2018. Specifically, we calculated Spearman (ρ) correlation coefficients to assess and compare the strength and consistency of the relationships between the daily model-derived estimates, and AQS, IMPROVE, WRCC and AIRSIS observation network data for the coterminous United States. We assessed correlations between the measurement and model data for location of monitor sites, and the following timescales: yearly, monthly, and wildfire activity periods. We stratified the model-derived and monitor-derived estimates of daily PM_2.5_ concentrations into bins of <35 μg/m^3^, 35–70 μg/m^3^, and >70 μg/m^3^ PM_2.5_ to assess the strength of the associated between the estimates over a wide-range of smoke concentrations. We classified Arizona, California, Colorado, Idaho, Montana, Nevada, New Mexico, Oregon, Utah, Washington, and Wyoming as “high wildfire impact states,” aggregated the monitor- and model-derived estimates for counties in these states, and assessed correlations between the monitor- and model-derived estimates. We used the Center for Disease Control and Prevention National Center for Health Statistics (NCHS) Urban-Rural Classification Scheme for Counties (Ingram and Franco, 2014) to estimate urban-rural differences in smoke impacts. For this analysis, we reclassified the 2013 NCHS urban-rural classifications from six categories to three, by grouping large central metropolitan counties and large fringe metropolitan counties together as large metropolitan counties, medium metropolitan counties and small metropolitan counties as medium metropolitan or small metropolitan counties, and micropolitan counties and non-core counties as non-metropolitan counties. We aggregated the counties into climatically consistent regions using the National Center for Environmental Information (NCEI) United States climate region classification (Karl and Koss, 1984) and examined spatial trends in the monitor networks, and model-derived estimates. Finally, we calculated county-level number of high PM_2.5_ days and person-days of exposure. We defined a high PM_2.5_ day as a day with daily mean PM_2.5_ concentration above 35 μg/m^3^ and calculated person-days of exposure as the sum of high PM_2.5_ days multiplied by the county population. We used county-level population data from the U.S. Census Bureau (2020) to calculate county-level person-days of exposure. Data analysis was performed using SAS version 9.4 (SAS Institute, Inc., Cary, North Carolina) and R software [R version 4.0.0 (2020-04-24), (R.Core Team, 2020).

## 3. Results

### 3.1. Descriptive statistics

**Fig. 1.**
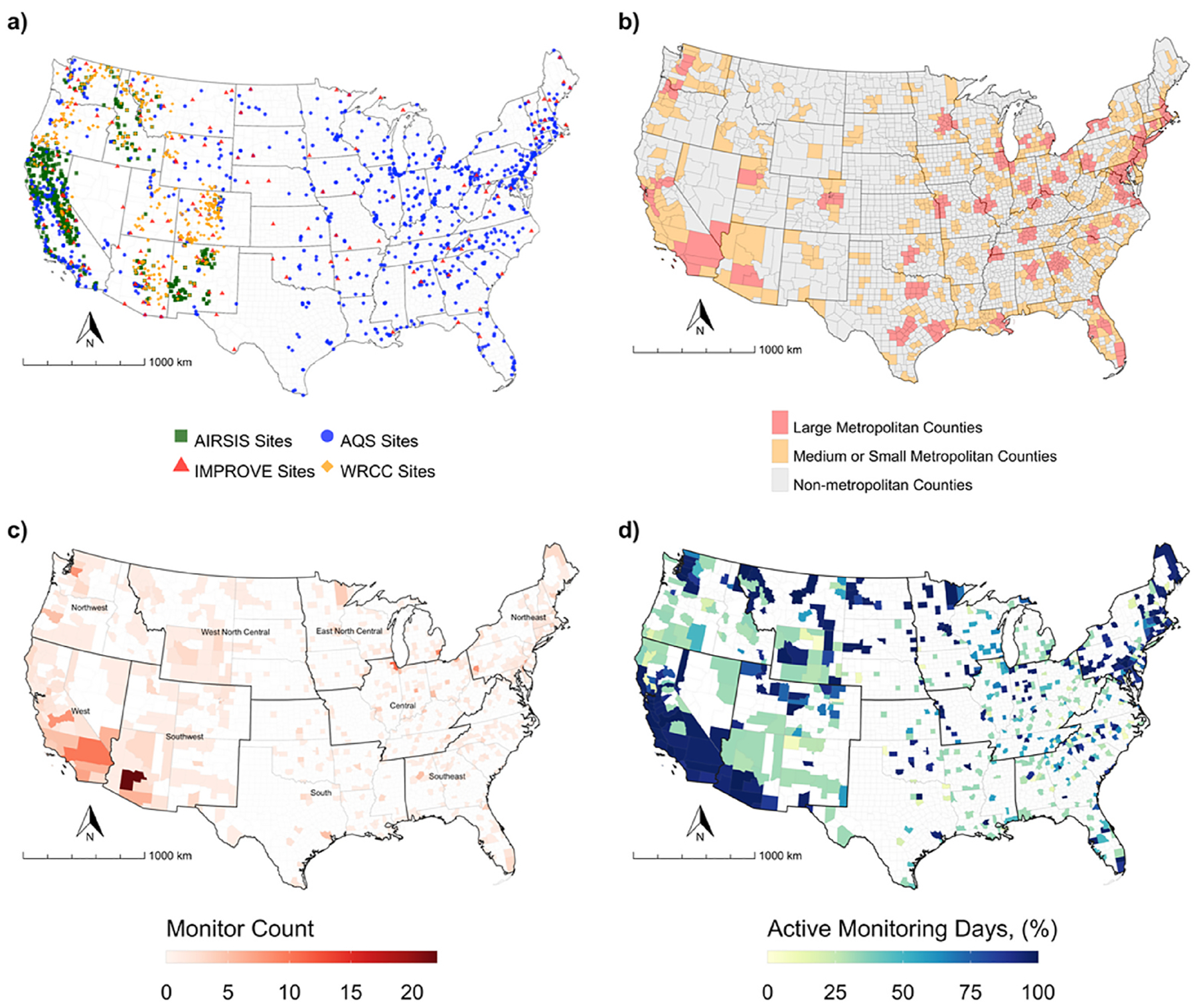
Distributions of monitors (panel a), counties in NCHS urban-rural classification categories (panel b), county-level numbers of AQS and IMPROVE monitors (panel c), and active PM_2.5_ monitoring days across the coterminous United States and over the 2015–2018 study period. Active monitoring days do not include measurements from temporary monitors (AIRSIS and WRCC).

AQS monitoring sites are predominantly located in large and medium metropolitan or small metropolitan counties, while IMPROVE, WRCC, and AIRSIS monitors are located in non-metropolitan counties (Fig. 1, panels a and b, Table 1). A similar spatial distribution is observed in the percentage of active monitoring days for permanent monitors in the coterminous United States across all observation years (Fig. 1, panel d). Overall, the west, southwest, and northeast regions had a greater porportion of counties that reported 60% or more active monitoring days over the observation period. The highest number of permanent monitors were observed in the west, central, southeast, and northeast regions of the domain, closely aligning with areas of high population density. Temporary monitors were predominantly located in the western regions, specifically northwest (Table 1).

### 3.2. Spatio-temporal trends in county-level PM_2.5_ concentrations

**Fig. 2.**
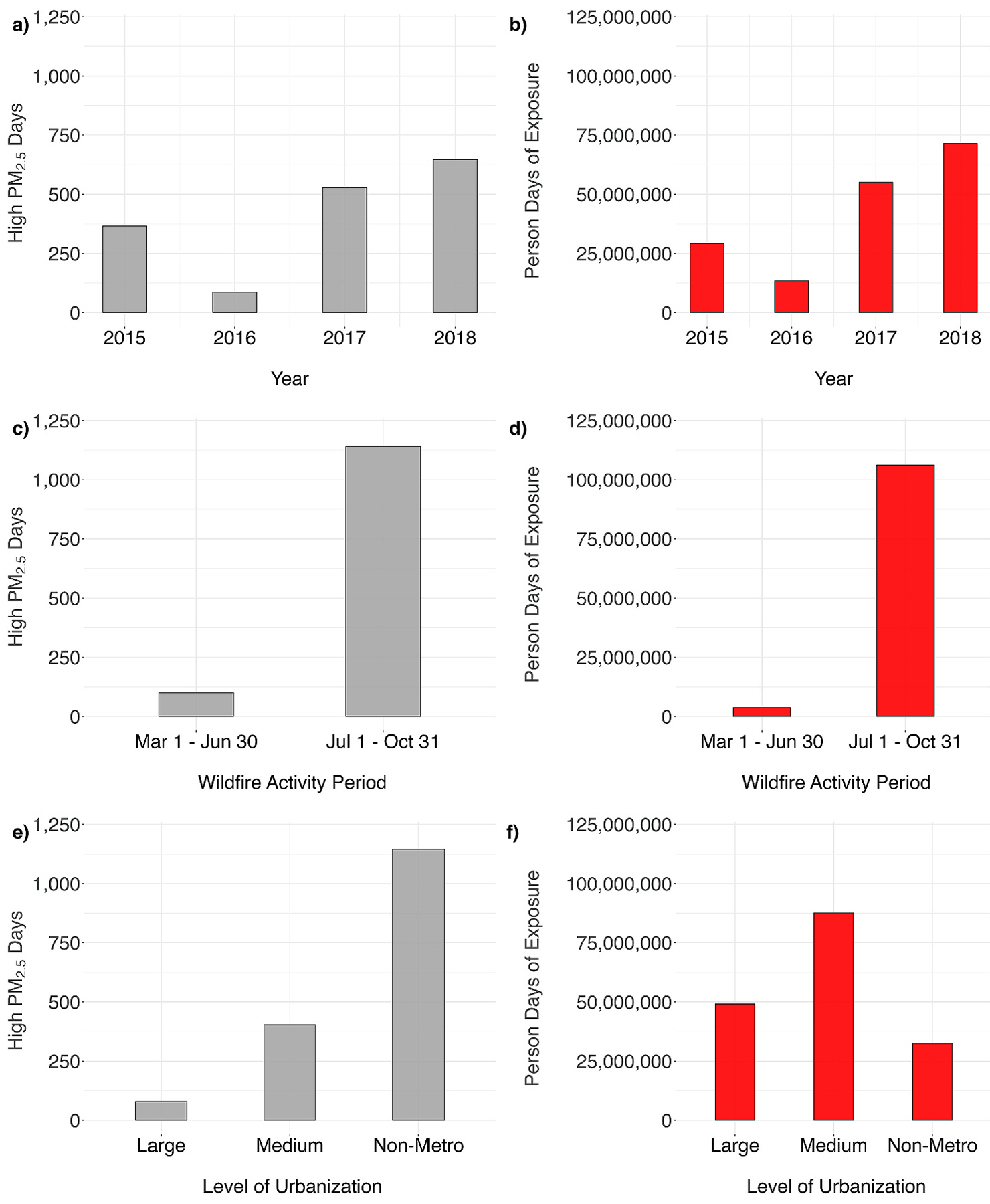
County-level model-derived high PM_2.5_ days (grey bars) and person-days of exposure (red bars) in high-wildfire impact states (AZ, CA, CO, ID, MT, NM, NV, OR, UT, WA, WY), by year (panels a and b), wildfire activity period (panel c and d), and level of urbanization (panel e and f). Large = large metropolitan counties, Medium = medium or small metropolitan counties, and Non-metro = Non-metropolitan counties.

**Fig. 3.**
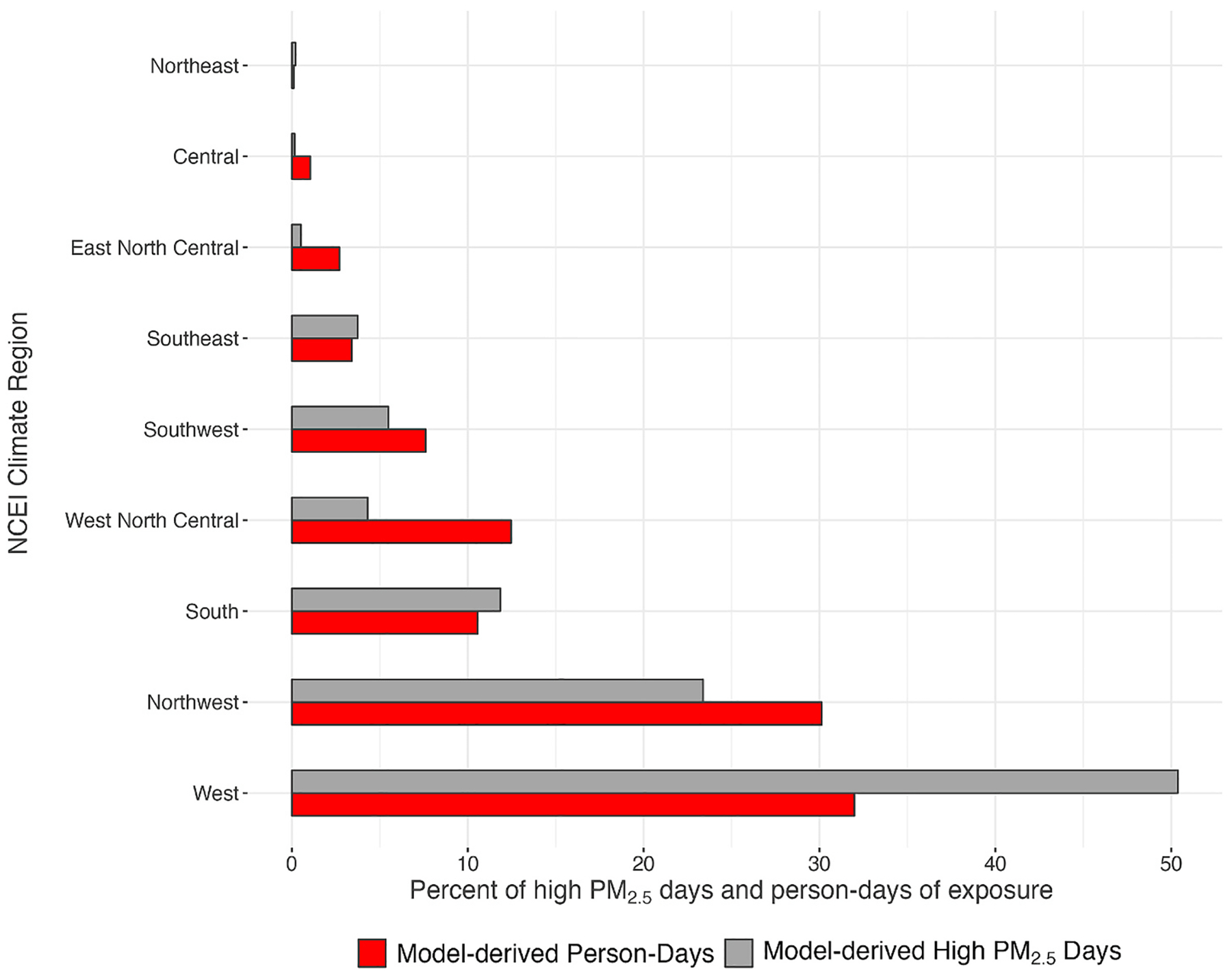
Estimates of model-derived person-days of exposure and high PM_2_._5_ days, by NCEI climate region.

**Fig. 4.**
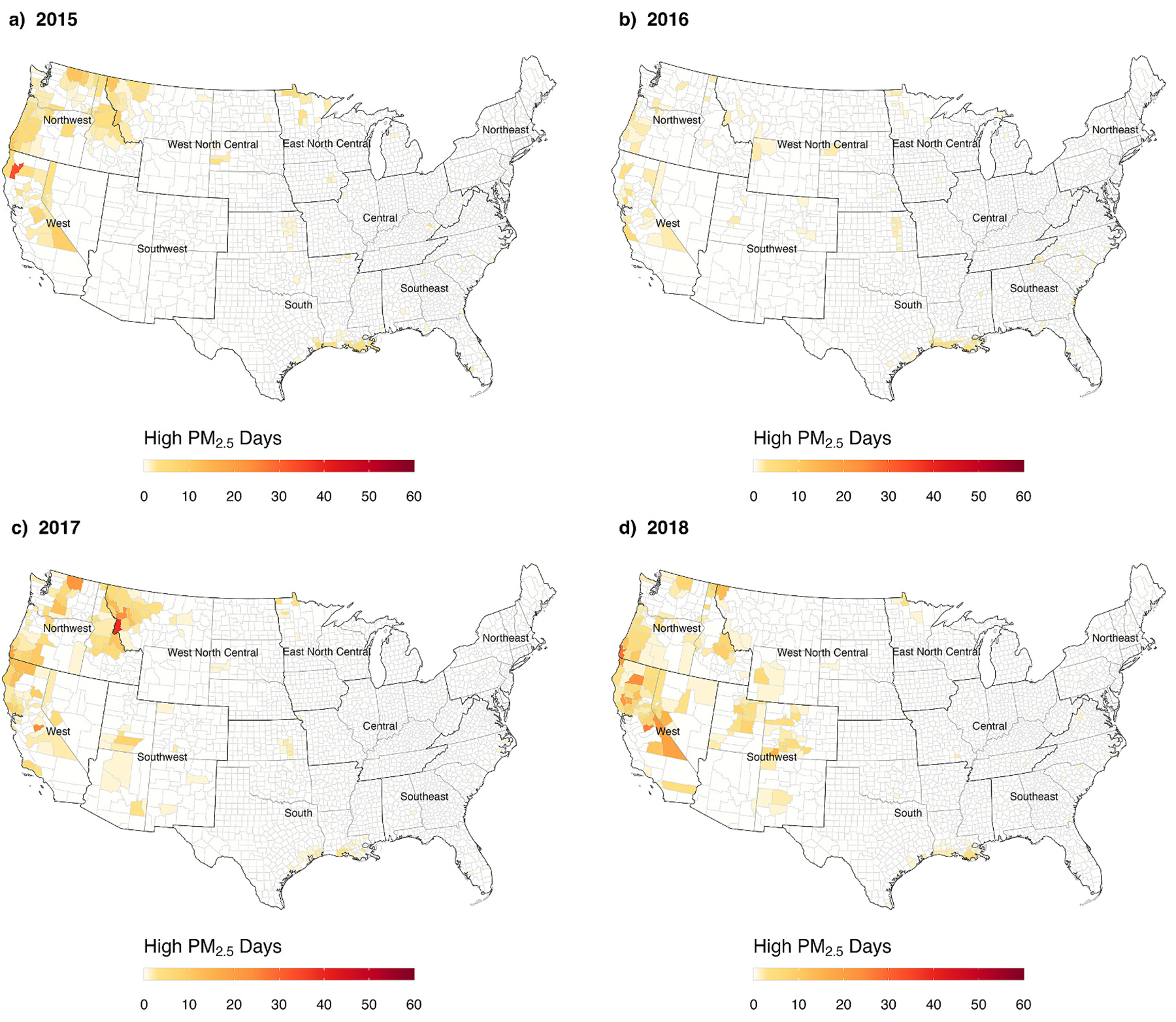
County-level estimates of the number of high PM_2.5_ days, defined as days when the daily mean PM_2.5_ concentration >35 μg/m^3^, for all counties for 2015 (panel a), 2016 (panel b), 2017 (panel c), 2018 (panel d).

When assessed spatially for years 2015–2018, counties with comparatively large numbers of high PM_2.5_ days were predominantly located in the west and southwest regions (in the northwest, and Northern and Southern California), highlighting contributions from large wildfire events in the region over the interval (Fig. 4 and S2). When aggregated temporally by wildfire activity periods, higher county-level high PM_2.5_ days and person-days were observed during the interval July 1–October 31 when compared the other intervals, and higher numbers of high PM_2.5_ days in the southwest and northwest regions (Fig. 2, panels c and d, and Fig. S1). We observed fewer high PM_2.5_ days in counties classified as large metropolitan, a larger number of county-level high PM_2.5_ days from counties classified as medium metropolitan or small metropolitan, with the largest contribution of county-level high PM_2.5_ days from counties classified as non-metropolitan (Fig. 2, panels e and f). Overall, counties in the northwest, south, west, and west north central climate regions accounted for over 70% of county-level high PM_2.5_ days and person-days of exposure (see Fig. 3).

### 3.3. Correlational analysis

When compared across all counties in the coterminous United States, and for all days, the relationship between daily county-level AQS- and model-derived estimates was positive but weak (ρ = 0.14, p < 0.001) (Table 2). A slightly stronger relationship was observed when comparing the model-derived estimates with IMPROVE-derived estimates (ρ 0.19, p < 0.001). When stratified by level of urbanization, correlations between the model- and AQS-derived estimates were positive but weak overall, increasing with decreasing levels of urbanization. This observation was also true when comparing model-derived estimates with IMPROVE-derived estimates, however, the relationship was slightly stronger for non-metropolitan counties (ρ = 0.23, p < 0.001). Correlations between the model- and IMPROVE-derived estimates showed a weak but positive relationship that increased with increasing PM_2.5_ concentrations. When aggregated by month, we observed stronger positive relationships over the months March–October and weaker positive relationships over the months November–February for both AQS- and IMPROVE-derived estimates (Table S1).

Correlations between model-derived and AQS- and IMPROVE-derived estimates of PM_2.5_ for counties in “high wildfire impact states” were positive, and overall displayed stronger relationships across all stratifications (level of urbanization, concentration ranges), and temporal aggregations (wildfire activity period, year, month) (Table 2 and S1). For example, stronger positive correlations were observed between model-derived and IMPROVE-derived estimates for counties in “high wildfire impact states” during August (ρ = 0.47, p < 0.001), as compared to all other months. Similarly, yearly and monthly comparisons for this region were relatively better (Table S1) and stratifying by concentration thresholds and levels of urbanization generated similar trends, but with relatively better correlations with the IMPROVE-derived than the AQS-derived estimates. Correlations were stronger across all spatial and temporal aggregations when the model-derived estimates were assessed with WRCC- and AIRSIS-derived estimates, compared to estimates from the permanent monitoring networks (Table 2). For example, the correlation between the model-derived estimates and the monitor-derived estimates for non-metropolitan counties increased when going from permanent to temporary networks (AQS: ρ = 0.25, IMPROVE: ρ = 0.29, WRCC: ρ = 0.47, AIRSIS: ρ = 0.54). Correlations between daily county-level model-derived, and AQS- and IMPROVE-derived estimates of PM_2.5_, resolved temporally for wildfire activity and spatially for counties in “high wildfire impact states,” improved slightly from the correlation observed for all counties in the coterminous United States (Table 2). The increase in the strength of the correlations was greater when comparing with the temporary monitoring network. Overall, stronger relationships were observed during July 1 to October 31 wildfire activity period, as compared to the March 1 to June 30 wildfire activity period. Finally, when stratified by levels of urbanization, we observed positive correlations between model- and monitor-derived PM_2.5_ estimates in “high wildfire impact states”, specifically in non-metropolitan counties.

## 4. Discussion

Overall, we observed relatively stronger positive correlations between county-level model- and monitor-derived estimates of PM_2.5_ concentrations in non-metropolitan areas and at higher concentration thresholds, and poorer performance for dense urban areas and lower concentration thresholds. This was especially true when compared with estimates from the temporary monitoring network which are primarily situated in non-metropolitan areas. Relatively stronger correlations were observed between model- and monitor-derived estimates for the temporary network when compared to the permanent network across all stratifications (level of urbanization, concentration), and temporal aggregations (wildfire activity period, year, month). This result highlights the inability of the permanent monitors to adequately characterize population exposure to wildfire smoke, especially in non-metropolitan counties, and was reported elsewhere (Larkin 2019; Jaffe et al., 2020).

The model data adequately describe the spatial distribution of the county-level PM_2.5_ concentrations, especially when assessed using temporary network data. County-level high PM_2.5_ days and person-days of exposure across all spatial and temporal aggregations displayed similar trends in the model-derived data as seen in the monitoring network data. We did observe that the model data were unable to accurately estimate county-level PM_2.5_ concentrations, especially at higher concentration, measured by monitoring data. Following their validation of the BlueSky modeling framework, Larkin et al. (2009) reported that the model adequately reproduces plume shape and long-range transport, but underpredicts near-field ground concentrations such as those within 100 km, and this observation aligns with the spatial trends and relationships shown in our analysis. It must be noted that the model-derived PM_2.5_ smoke estimates only include information regarding wildfire emissions. When compounded with the meteorological processes governing smoke transport to observation sites, we expect the correlations between the model-derived estimates and the monitor-derived estimates to be underestimated at higher concentrations.

Furthermore, climate change significantly affects the occurrence and severity of wildfires (Abatzoglou and Williams, 2016; De Groot et al., 2013; Westerling et al., 2006; Westerling and Bryant, 2008), the length of fire seasons (De Groot et al., 2013; Flannigan et al., 2013), and the total area burned (Gillett et al., 2004). Westerling et al. (2006) examined associations between climate-related temperature increases and changes in wildfire frequency in the western United States and found strong correlations between regional temperatures and interannual variability in wildfire frequency during early and peak wildfire seasons. According to Westerling et al. (2006), the average length of the wildfire season increased by 64% between 1987 and 2003 when compared to the average over the period 1970 to 1986. This increase in the frequency and magnitude of wildfire episodes underscores the importance of smoke forecast products to characterize wildfire exposures in communities, especially those residing in the wildland-urban interface.

Consideration should be given to certain limitations that exist for smoke forecast products, particularly around their inputs, which can significantly affect the forecasts generated. Studies have shown that difficulties in characterization of emissions, plume rise, chemistry, transport, coupled atmosphere and smoke mechanisms, and the choice of fire information are major drivers of uncertainties the models (Larkin et al., 2009, Jaffe et al., 2020; Larkin et al., 2012; Raffuse et al., 2012). Studies assessing the performance of smoke forecasting system have reported large model overestimation bias attributed to chemistry representation (Baker et al., 2016), and significant sensitivity to small errors in geolocation of fires and vertical distribution of emissions (Garcia-Menendez et al., 2014). While these limitations exist in the ability of forecast products to accurately assess ground-level concentrations, these predictions have utility in identifying areas and populations exposed to wildfire smoke. However, the concentration levels at which populations are exposed might be misrepresented and may lead to misclassification of smoke exposure in these populations.

## 5. Conclusions

The development of effective strategies to minimize adverse effects of wildfire smoke requires the availability of data that can reliability identify wildfires, characterize PM_2.5_ smoke concentrations at adequate spatio-temporal scales, and assess population-level exposures and health risks to wildfire-related PM. Our findings suggest that the model-derived data are better suited to identify communities that are impacted by heavy smoke events, especially during emergency response and for communities located near wildfire episodes; however, public health practitioners and health researchers need to consider the limitations associated with modeled data products before using these predictions to conduct epidemiologic research, especially for assessments that require a more rigorous smoke exposure characterization before, during, and after heavy smoke events.

## Supplementary Material

public health applications of historical smoke forecast

## Figures and Tables

**Table 1:** Characteristics of AQS, IMPROVE, WRCC, and AIRSIS networks by level of urbanization and NCEI climate region.

Spatial Aggregation	Spatial Aggregation Category	Counties	Monitors		Counties With At Least One Monitor^c^		Population Covered By Monitors^d^, %	Temporary Monitors	
			AQS^a^	IMP^b^	AQS	IMP		WRCC^e,g^	AIRSIS^f,g^
Level of Urbanization	Large metropolitan counties	436	385	20	176	16	83	119	417
	Medium or small metropolitan counties	725	376	42	272	35	69	221	560
	Non-metropolitan counties	1948	183	96	160	89	17	409	919
NCEI Climate Region	Central	666	184	8	117	8	62	–	–
	East North Central	341	91	8	62	8	62	–	–
	Northeast	245	164	16	101	15	75	–	–
	Northwest	119	41	16	29	15	73	221	120
	South	658	80	10	57	10	52	–	–
	Southeast	573	132	20	106	15	61	10	6
	Southwest	141	66	36	37	29	86	368	243
	West	75	123	22	48	19	99	22	1490
	West North Central	291	63	22	51	21	60	128	30

a EPA Air Quality System (AQS).

b Inter-agency Monitoring of Protected Visual Environments (IMPROVE). Elemental carbon fraction of total PM_2.5_ mass used to evaluate model data.

c Does not account for temporary monitors.

d Fraction of population in spatial aggregation covered by at least one monitor from permanent monitoring networks.

e Western Region Climate Centers (WRCC).

f Interagency Real Time Smoke Monitoring (AIRSIS).

g No comparisons for some climate regions using the WRCC and AIRSIS networks because these monitors are only located in the western United States.

**Table 2:** Correlation between county-level model-derived and AQS-, IMPROVE-, WRCC-, and AIRSIS-derived estimates of PM_2.5_ concentrations.

Spatial Aggregation	Stratification		Model vs AQS^a^ ρ (p-value)	Model vs IMP^b^ ρ (p-value)	Model vs WRCC^c,e^ ρ (p-value)	Model vs AIRSIS^d.e^ ρ (p-value)
Coterminous United States	All Observations		0.14 (<0.001)	0.19 (<0.001)	–	–
	Level of Urbanization	Large metropolitan counties	0.10 (<0.001)	0.08 (<0.001)	–	–
		Medium or small metropolitan counties	0.16 (<0.001)	0.17 (<0.001)	–	–
		Non-metropolitan counties	0.18 (<0.001)	0.23 (<0.001)	–	–
	Concentration Bins	<35 μg m^− 3^	0.13 (<0.001)	0.18 (<0.001)	–	–
		35–70 μg m^− 3^	0.11 (=0.034)	0.20 (=0.05)		
		>70 μg m^− 3^	0.21 (<0.001)	NS	–	–
	Wildfire Activity Period	March 1-June 30	0.17 (<0.001)	0.21 (<0.001)		
		July 1-October 31	0.15 (<0.001)	0.21 (<0.001)		
High Wildfire Impact States: AZ, CA, CO, ID, MT, NM, NV, OR, UT, WA, WY	All Observations		0.18 (<0.001)	0.25 (<0.001)	0.43 (<0.001)	0.43 (<0.001)
	Level of Urbanization	Large metropolitan counties	0.11 (<0.001)	0.10 (<0.001)	0.08 (<0.001)	0.18 (<0.001)
		Medium or small metropolitan counties	0.22 (<0.001)	0.23 (<0.001)	0.41 (<0.001)	0.29 (<0.001)
		Non-metropolitan counties	0.25 (<0.001)	0.29 (<0.001)	0.47 (<0.001)	0.54 (<0.001)
	Concentration Bins	<35 μg m^− 3^	0.18 (<0.001)	0.24 (<0.001)	0.39 (<0.001)	0.37 (<0.001)
		35–70 μg m^− 3^	0.15 (=0.006)	0.22 (=0.04)	NS	NS
		>70 μg m^− 3^	0.22 (<0.001)	NS	NS	0.22 (<0.001)
	Wildfire Activity Period	March 1 to June 30	0.21 (<0.001)	0.20 (<0.001)	0.27 (<0.001)	0.22 (<0.001)
		July 1 to October 31	0.26 (<0.001)	0.34 (<0.001)	0.54 (<0.001)	0.47 (<0.001)

AZ = Arizona, CA = California, CO = Colorado, ID = Idaho, MT = Montana, NM = New Mexico, NV = Nevada, OR = Oregon, UT = Utah, WA = Washington, WY = Wyoming.

NS = not significant.

a EPA Air Quality System (AQS).

b Inter-agency Monitoring of Protected Visual Environments (IMPROVE). Elemental carbon fraction of total PM_2.5_ mass used to evaluate model data.

c Western Region Climate Centers.

d Interagency Real Time Smoke Monitoring.

e No comparisons for the coterminous United States using the WRCC and AIRSIS networks because these monitors are only located in the western United States.
