# Supplementary material for: Public health applications of historical smoke forecasts: An evaluation of archived BlueSky data for the coterminous United States, 2015–2018
Source: Comput Geosci. Author manuscript; Available in PMC 2024 Aug 2. (PMC11296727; doi:10.1016/j.cageo.2022.105267)
Supplement: public health applications of historical smoke forecast [file NIHMS2012331-supplement-public_health_applications_of_historical_smoke_forecast.docx]

**Public Health Applications of Historical Smoke Forecasts: An Evaluation of Archived BlueSky data for the Coterminous United States, 2015- 2018**

**Ryan Michael, PhD; Maria C. Mirabelli, PhD; and Ambarish Vaidyanathan, PhD;**

Climate and Health Program, National Center for Environmental Health, Centers for Disease Control and Prevention, 4770 Buford Highway NE, Mailstop S106-6, Atlanta, GA 30341 US

Manuscript Reference Number: CAGEO_CAGEO-D-20-00455

**Supplementary Data**

Table S1. Correlation between county-level model-derived and AQS-, IMPROVE-, WRCC-, and AIRSIS-derived estimates

| Spatial Aggregation | Temporal  Aggregation | | Model vs AQS^1^  ρ (p-value) | Model vs IMPROVE^2^  ρ (p-value) | Model vs WRCC^3,5^  ρ (p-value) | Model vs AIRSIS^4,5^  ρ (p-value) |
| --- | --- | --- | --- | --- | --- | --- |
| Coterminous United States | Year | 2015 | 0.09 (<0.001) | 0.15 (<0.001) | - | - |
|  |  | 2016 | 0.14 (<0.001) | 0.18 (<0.001) | - | - |
|  |  | 2017 | 0.14 (<0.001) | 0.21 (<0.001) | - | - |
|  |  | 2018 | 0.17 (<0.001) | 0.21 (<0.001) | - | - |
|  | Month | January | 0.04 (<0.001) | 0.17 (<0.001) | - | - |
|  |  | February | 0.10 (<0.001) | 0.21 (<0.001) | - | - |
|  |  | March | 0.24 (<0.001) | 0.28 (<0.001) | - | - |
|  |  | April | 0.22 (<0.001) | 0.23 (<0.001) | - | - |
|  |  | May | 0.12 (<0.001) | 0.16 (<0.001) | - | - |
|  |  | June | 0.11 (<0.001) | 0.15 (<0.001) | - | - |
|  |  | July | 0.09 (<0.001) | 0.13 (<0.001) | - | - |
|  |  | August | 0.21 (<0.001) | 0.34 (<0.001) | - | - |
|  |  | September | 0.16 (<0.001) | 0.20 (<0.001) | - | - |
|  |  | October | 0.20 (<0.001) | 0.19 (<0.001) | - | - |
|  |  | November | 0.16 (<0.001) | 0.12 (<0.001) | - | - |
|  |  | December | 0.09 (<0.001) | 0.06 (<0.001) | - | - |
| High Wildfire Impact States: AZ, CA, CO, ID, MT, NM, NV, OR, UT, WA, WY | Year | 2015 | 0.14 (<0.001) | 0.20 (<0.001) | 0.42 (<0.001) | 0.38 (<0.001) |
|  |  | 2016 | 0.16 (<0.001) | 0.20 (<0.001) | 0.29 (<0.001) | 0.21 (<0.001) |
|  |  | 2017 | 0.18 (<0.001) | 0.27 (<0.001) | 0.50 (<0.001) | 0.49 (<0.001) |
|  |  | 2018 | 0.24 (<0.001) | 0.29 (<0.001) | 0.45 (<0.001) | 0.53 (<0.001) |
|  | Month | January | 0.02 (<0.001) | 0.07 (<0.001) | NS | NS |
|  |  | February | 0.10 (<0.001) | 0.12 (<0.001) | NS | NS |
|  |  | March | 0.13 (<0.001) | 0.13 (<0.001) | NS | 0.20 (<0.001) |
|  |  | April | 0.10 (<0.001) | 0.17 (<0.001) | 0.22 (<0.001) | 0.17 (<0.001) |
|  |  | May | 0.09 (<0.001) | 0.19 (<0.001) | 0.17 (<0.001) | 0.10 (<0.001) |
|  |  | June | 0.18 (<0.001) | 0.25 (<0.001) | 0.39 (<0.001) | 0.22 (<0.001) |
|  |  | July | 0.23 (<0.001) | 0.28 (<0.001) | 0.43 (<0.001) | 0.50 (<0.001) |
|  |  | August | 0.33 (<0.001) | 0.47 (<0.001) | 0.49 (<0.001) | 0.50 (<0.001) |
|  |  | September | 0.24 (<0.001) | 0.34 (<0.001) | 0.49 (<0.001) | 0.47 (<0.001) |
|  |  | October | 0.21 (<0.001) | 0.26 (<0.001) | 0.18 (<0.001) | 0.33 (<0.001) |
|  |  | November | 0.21 (<0.001) | 0.21 (<0.001) | 0.10 (<0.001) | 0.44 (<0.001) |
|  |  | December | 0.09 (<0.001) | 0.07 (<0.001) | NS | NS |

**^1^**EPA Air Quality System (AQS)

^2^ Inter-agency Monitoring of Protected Visual Environments (IMPROVE). ). Elemental carbon fraction of total PM_2.5_ mass used to evaluate model data

^3^Western Region Climate Centers

^4^Inter-agency Real Time Smoke Monitoring Network

^5^No comparisons for the coterminous United States using the WRCC and AIRSIS networks because these monitors are only located in the western United States

AZ = Arizona, CA = California, CO = Colorado, ID = Idaho, MT = Montana, NM = New Mexico, NV = Nevada, OR = Oregon, UT = Utah, WA = Washington, WY = Wyoming

NS = not significant

No comparisons for the coterminous United States using the WRCC and AIRSIS networks because these monitors are only located in the western United States

**
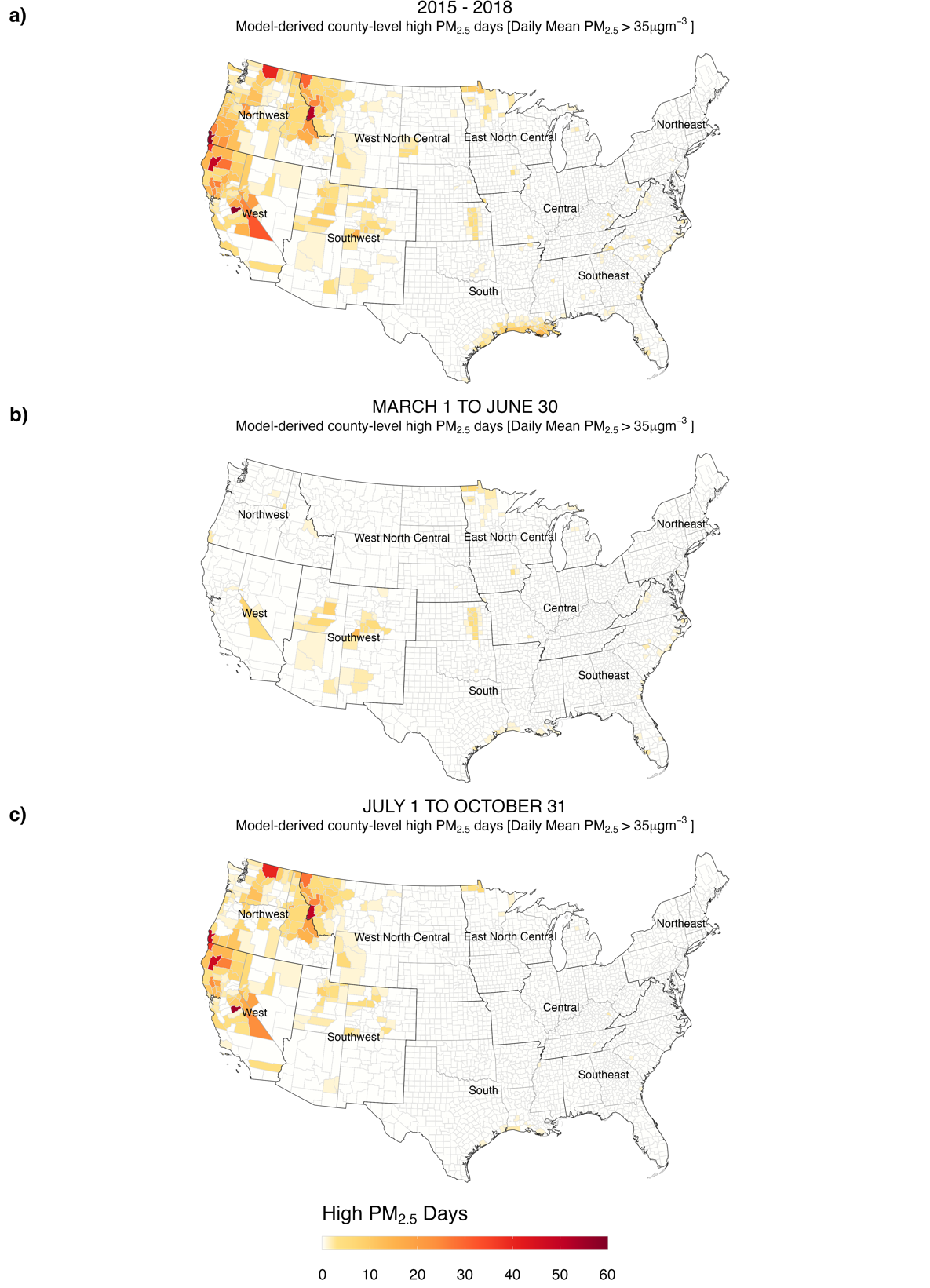
**

Figure S1. Spatio-temporal distribution of county-level estimates of model-derived PM_2.5_ concentrations. Data display model-derived estimates of county-level high PM_2.5_ days. High PM_2.5_ days were defined as days when the daily mean PM_2.5_ concentration is greater than 35 µg/m^3^. Panel a show data for all months over the study period (2015–2018), panel b shows data for period March 1 to June 30, and panel c shows data for period July 1 to October 31.

**
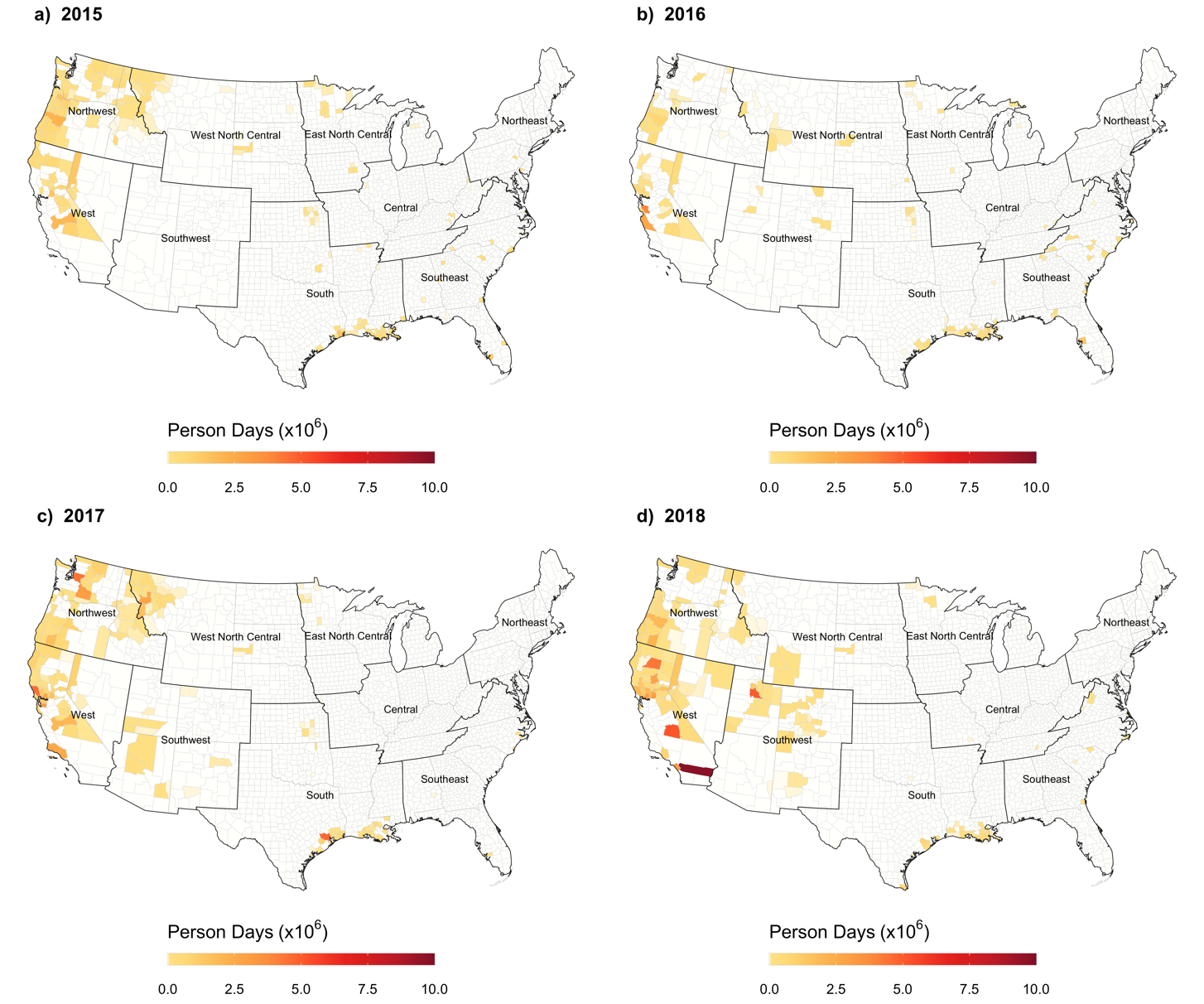
**

Figure S2. County-level person-days of exposure for model-derived estimates of PM_2.5_ concentrations for all counties for 2015 (panel a), 2016 (panel b), 2017 (panel c), and 2018 (panel d). Smoke days are defined as days when the daily mean PM_2.5_ concentration is greater than 35 µg/m^3^.
